# Completely ulcerated infantile hemangioma: a diagnostic challenge

**DOI:** 10.1007/s00508-025-02543-5

**Published:** 2025-05-22

**Authors:** Elias Marquart, Doris Weiss, Klaudija Batinic, Thomas Wiesner, Johannes Rohrbeck, Tamara Arnoldner, Wolfgang Weninger, Tamar Kinaciyan

**Affiliations:** 1 Department of Dermatology, Medical University of Vienna, Währinger Gürtel 18–20, 1090 Vienna, Austria; 2 St. Anna Children’s Hospital Vienna, Vienna, Austria; 3 Clinical Institute of Pathology, Medical University of Vienna, Vienna, Austria

**Keywords:** Systemic propranolol treatment, Rapid treatment response, Histology, Immunohistochemistry, Glucose transporter 1 staining

## Abstract

The present case report describes a completely ulcerated infantile hemangioma (UIH) in a 5-month-old infant on the left proximolateral thigh initially misdiagnosed as pyoderma gangrenosum, sporotrichosis or atypical mycobacterial infection. Clinical assessment, histological findings, and GLUT‑1 immunohistochemistry confirmed the diagnosis of UIH. Systemic propranolol treatment led to rapid ulcer healing within 3 weeks and complete recovery without relapse after 18 months of treatment. The report emphasizes the diagnostic challenges, effective propranolol treatment and the importance of considering UIH in the differential diagnoses of solitary pediatric ulcers.

## Introduction

Infantile hemangioma (IH) is a prevalent vascular tumor in infancy. A potential complication is ulceration, particularly in the thigh and the anogenital regions. When complete ulceration occurs, misdiagnosis may lead to prolonged disease duration and delayed initiation of treatment. Therefore, ulcerated infantile hemangioma (UIH) should be considered as a differential diagnosis in pediatric patients with solitary ulcers. This case report emphasizes the clinical course of a UIH initially misdiagnosed as pyoderma gangrenosum, sporotrichosis or atypical mycobacterial infection, ultimately successfully treated with systemic propranolol.

## Case report

**Fig. 1:**
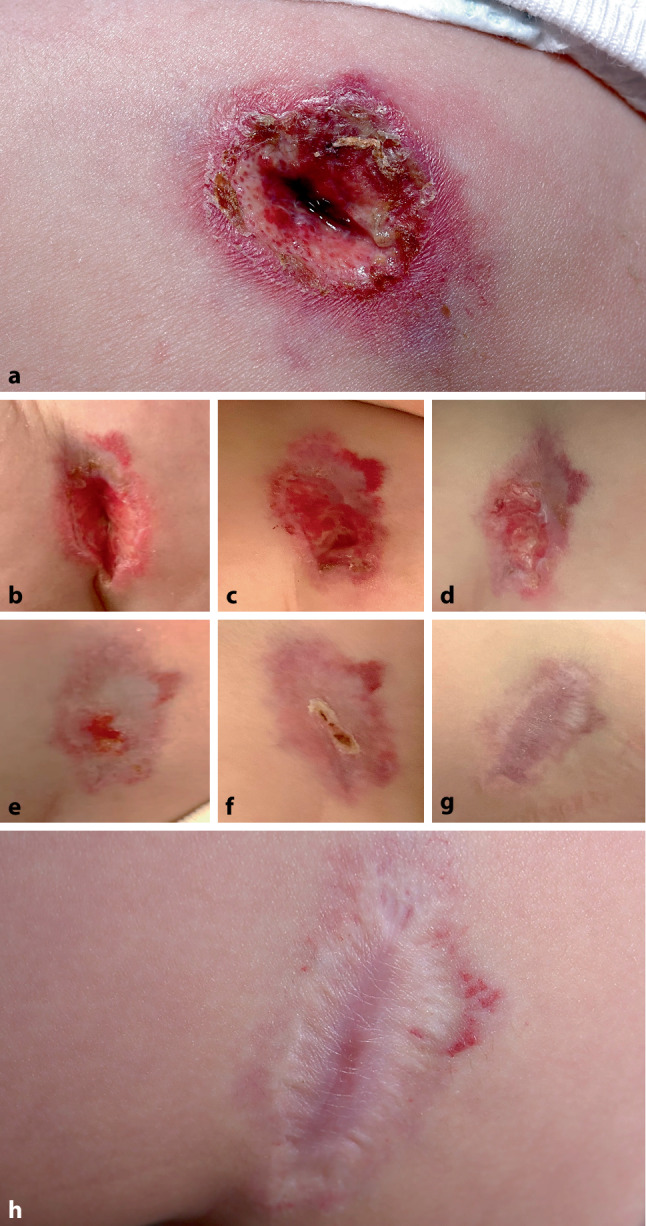
Course of propranolol treatment of the ulcer (1.5 cm deep and 5.0 cm in diameter) in a 5-,month-old infant. **a** Initial presentation, **b** day 6, **c** day 11, **d** day 16, **e** day 20, **f** day 24, **g** day 70, **h** day 266

**Fig. 2:**
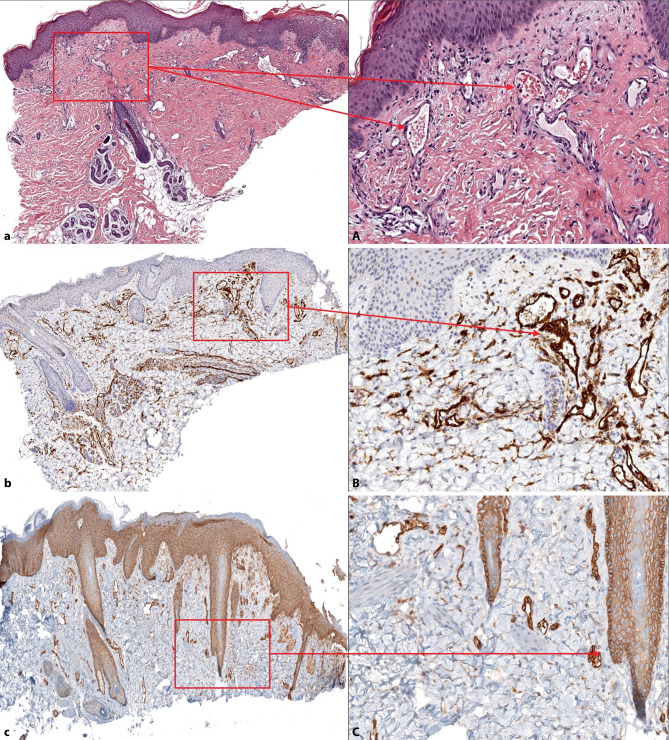
Histology and immunohistochemistry. Left column shows always a magnification of 40 x, right column a magnification of 200 x detail from the marked part of the left picture. **a**) Histology, H&E staining shows an increased number of dilated vessels and mild fibrosis. **A**) Histology shows dilated vessels with accumulated erythrocytes in detail. **b**) Immunochemistry, CD31 staining highlights the normal vascular endotheilial cells. **B**) CD31 staining in detail. **c**) GLUT‑1 staining shaws a positive reaction of endothelial cells, confirming the diagnosis of involuting infantile hemangioma. **C**) again an excerpt from **c**)

An age-appropriately developed 5‑month-old infant was referred within our department to the pediatric dermatology outpatient clinic due to an ulcer on the upper left side of the thigh. The ulcer developed 1 month ago, 2 weeks after the patient returned from Turkey at 3 months of age (Fig. 1a). The ulcer continued to grow in size and depth despite prior treatment with topical povidone-iodine and steroids prescribed by in-office pediatricians and dermatologists. Previous differential diagnoses considered at the first visit in our general dermatology department included pyoderma gangrenosum, sporotrichosis and atypical mycobacterial infection. Microbiological testing (bacterial/fungal culture, Leishmania PCR) was performed and yielded negative results. Dermatopathological evaluation obtained from a 4mm punch biopsy of the border of the lesion showed only increased numbers of dilated vessels with erythrocytes and mild fibrosis without other abnormal findings in hematoxylin-eosin staining (Fig. 2a, A).

The dermatological examination at the pediatric dermatology clinic revealed a painful fibrin-coated ulceration approximately 1.5 cm in depth and 5.0 cm in diameter with scaly border and surrounding erythema on the proximal left anterolateral thigh without active bleeding leading to the clinical diagnosis of an ulcerated infantile hemangioma. Upon further inquiry, the mother described a prior lesion as a red spot at the ulcer site, first noticed when the infant was 3 months old. No photograph of the initial lesion was available.

The infant was admitted to hospital for careful initiation of systemic beta-blocker treatment under cardiorespiratory monitoring. Treatment with oral weight-adapted propranolol (1 mg/kg) twice daily as well as concomitant wound care and analgesia as needed were established. The patient could be discharged 2 days later with this dosage to homecare as the treatment was well tolerated without any side effects. The ulcer started to heal rapidly and complete wound healing was achieved within 3 weeks (Fig. 1b–g). Propranolol treatment was continued for 18 months in total. An annual check-up 1 year after the end of treatment showed a smooth and painless scar without any signs of relapse. Additionally, we performed immunohistochemical staining with Ki67, a proliferation marker in tumors, CD31 staining, a marker for normal vascular endothelial cells, CD34 staining, a marker for immature endothelial cells and glucose transporter 1 (GLUT-1) staining, a marker for infantile endothelial cells.

Immunohistochemical staining with Ki67 was negative (data not shown). The CD31 staining for normal vascular endothelial cells (Fig. 2b, B) and CD34 staining for immature endothelial cells (data not shown) were both comparably positive. Finally, GLUT‑1 staining for infantile endothelial cells was positive as well (Fig. 2c, C), confirming our initial clinical diagnosis.

## Discussion

With approximately 4% of infants affected, IH is a common pediatric vascular tumor entity with reported prevalence rates ranging from 2% to 10% in the literature [1]. Risk factors such as low birth weight, prematurity and female sex are associated with higher disease prevalence. The pathogenesis of IH still remains to be elucidated but the natural clinical course can be divided into a proliferative and involutional phase. The IH-related complications are body region-dependent and include physical impairment, disfigurement and bleeding in cases of prior ulceration [1]. Ulceration occurs in approximately 16% of all IH during the proliferative phase and often shows concurrent bleeding [2]. Moreover, IHs localized within the diaper area and head region are more prone to ulceration than other anatomical regions [3]; however, complete ulceration without vascular remnants is rarely described in literature [4]. Misdiagnosis within the pelvic region as juvenile xanthogranuloma, fibrosarcoma, rhabdomyosarcoma or kaposiform hemangioendothelioma may occur as these conditions can exhibit clinical features similar to IH [5, 6]. Treatment options for IH include topical or systemic beta-blockers, laser treatment (Nd:YAG, PDL) and surgery whereas early referral and implementation of systemic propranolol treatment has been proven to be safe and effective in IH with largely varying mean healing times ranging from 5 days to 8 months [7, 8]. Moreover, in uncomplicated IH initiation of propranolol treatment with low-dose regimens (≤ 1 mg/kg per day) is associated with a 2.04 times shorter healing time compared to higher dose (> 1 mg/kg per day) approaches [9]; however, in cases of ulceration, higher doses (2–3 mg/kg per day) are often prescribed and are considered the first-line treatment for complicated hemangiomas [10]. Additionally, evidence for optimal wound care is incongruent, with no superiority found for widely used silver-coated dressings; therefore, dressing selection should be patient-oriented and pain management should be prioritized during dressing changes [11].

In summary, the present case report of an UIH i) demonstrates the diagnostic challenge of completely ulcerated lesions without vascular remnants, ii) highlights the clinical course and rapid response to systemic propranolol treatment, precisely documented in pictures (Fig. 1a–h), iii) and depicts the histological and immunohistochemical diagnostic findings in UIH (Fig. 2c).

## Abbreviations

ESPD: European Society of Pediatric Dermatology
GLUT‑1: Glucose transporter 1
H&E: Hematoxylin and eosin
IH: Infantile hemangioma
Nd: YAG: neodymium-doped yttrium aluminium garnet
PCR: Polymerase chain reaction
PDL: Pulsed dye laser
UIH: Ulcerated infantile hemangioma
